# The overexpression of GPX8 is correlated with poor prognosis in GBM patients

**DOI:** 10.3389/fgene.2022.898204

**Published:** 2022-08-17

**Authors:** Sibo Li, Xudong Jiang, Meicun Guan, Yi Zhang, Yanfei Cao, Lina Zhang

**Affiliations:** ^1^ Departments of Laboratory Diagnosis, Daqing Oilfield General Hospital, Daqing, China; ^2^ Departments of Laboratory Diagnosis, The Second Affiliated Hospital of Jiamusi University, Jiamusi, China; ^3^ Departments of Laboratory Diagnosis, The Fifth Affiliated Hospital of Harbin Medical University, Daqing, China; ^4^ Harbin Medical University (Daqing), Daqing, China

**Keywords:** GPX8, GBM, PMT, immune infiltration, prognosis

## Abstract

Glutathione peroxidase 8 (GPX8), located in the endoplasmic reticulum, is associated with poor prognosis in several cancers. However, the expression and functions of GPX8 in cancers remain unclear. The purpose of this study was to explore the expression and functions of GPX8 in glioblastoma (GBM). We obtained expression data of GPX8 by accessing the TCGA, CGGA, GEPIA, and TIMER2.0 databases and validated them using western blot and immunohistochemistry. The Kaplan–Meier overall survival curve and Cox regression model were used to evaluate the prognostic value of GPX8 in glioma patients. Gene ontology (GO) and function enrichment analysis were used to investigate the potential function of GPX8 in GBM. Correlation analysis was used to clarify the role of GPX8 in proneural–mesenchymal transition (PMT). We studied the correlation between GPX8 expression and GBM immune infiltration by accessing cBioPortal and TIMER2.0 databases. Here, we demonstrated that GPX8 was significantly upregulated in GBM, and was associated with IDH-wildtype and mesenchymal subtype with poor prognosis. Survival analysis results indicated that GPX8 is an independent prognostic factor for overall survival (OS) in all WHO-grade glioma patients. Through the functional studies, we found that high expression of GPX8 correlated with mesenchymal signature and negatively correlated with proneural signature, indicating that GPX8 might promote PMT in GBM. Finally, based on correlation analysis, we found that the expression of GPX8 was associated with immune infiltration and the IL1/MYD88/IRAK/NF-κB pathway in GBM. Our results show that GPX8 is a key factor affecting the prognosis of GBM patients, and its targeting has the potential to provide a novel therapeutic approach.

## Introduction

Glioma is the most common primary brain tumor, and about 100,000 people are diagnosed worldwide each year. High mortality and recurrence rate are well-known features of glioma (Bray et al., 2018). Glioblastoma (GBM) is a highly malignant glioma classified by the World Health Organization (WHO) as grade IV (Louis et al., 2016). The median survival time of GBM patients remains less than 2 years, and the 5-year survival rate remains less than 7% (Molinaro et al., 2019). During the course of the disease, GBM patients are often accompanied by malignant complications such as epilepsy and cerebral edema, which seriously affect the patient’s ability to live and have a devastating impact on their family. Despite the continuous development of various therapy methods such as surgery, chemotherapy, radiotherapy, and immunotherapy, they can only improve the survival of patients to a limited extent. In addition, GBM is characterized by extensive genetic heterogeneity, and some targeted therapies for traditional molecules, such as inhibition of EGFR and PI3K, do not benefit clinical GBM patients (Stupp et al., 2005; McNamara et al., 2014; Li et al., 2016; Westphal et al., 2017). Thus, exploring new therapeutic targets may provide new insights into GBM targeted therapy.

Glutathione peroxidase (GPx) combines with glutathione to decompose peroxides into alcohols (Tosatto et al., 2008), and plays an antioxidant function by removing reactive oxygen species (ROS) deposited in cells (Brigelius-Flohé and Maiorino, 2013). In addition to their antioxidant activity, GPx proteins also have significant effects on important physiological processes such as material metabolism and signal transduction (Peng et al., 2014; Guerriero et al., 2015; Milonski et al., 2015; Mrowicka et al., 2015; Huang et al., 2017). GPX8 is a type II transmembrane protein, which is the last confirmed member of the GPx family and has high sequence similarity with GPX7 (Brigelius-Flohé and Maiorino, 2013). GPX8 has multifarious functions in the body, which have been illustrated in recent studies. For example, GPX8 protects against endotoxic shock (Hsu et al., 2020), regulates oxidative stress during pregnancy (Mihalik et al., 2020), and maintains microsomal membrane lipid homeostasis in HELA cells (Bosello-Travain et al., 2020). In addition, GPX8 has also been found to promote tumor progression through multiple important pathways, such as Wnt and JAK/STAT3 (Chen et al., 2020; Khatib et al., 2020; Zhang et al., 2020). However, the tumor biological function of GPX8 remains unclear, especially in GBM.

In a recent study, the GBM subtypes were observed to transition from proneural to mesenchymal (Klughammer et al., 2018), resulting in increased tumor invasion and significantly reduced survival (Wu et al., 2020). Mesenchymal is the most aggressive subtype of GBM (Wang et al., 2019), and proneural–mesenchymal transition (PMT) can occur in GBM patients following drug therapy and radiotherapy (Segerman et al., 2016). Similar to epithelial–mesenchymal transition (EMT), the molecular mechanisms driving PMT elaborated the downregulation of CDH1 together with other proneural markers and upregulation of mesenchymal markers such as CDH2 (Fedele et al., 2019). Changes in cell phenotypes can affect sensitivity to radiotherapy and chemotherapy, which may be detrimental to some targeted therapies. Consequently, PMT can have significant effects on GBM patients and, as such, requires further exploration.

Here, we revealed the overexpression of GPX8 in GBM compared with normal brain samples, which has not been reported before. In addition, the upregulation of GPX8 was correlated with poor prognosis in high-grade glioma (HGG) patients. We further elucidated the potential mechanism of GPX8 involvement in GBM progression. At present, further laboratory studies are needed to explore the function of GPX8. However, our findings provide novel evidence for the involvement of GPX8 in the malignancy of GBM.

## Materials and methods

### Glioma and normal tissue samples

Central nervous system (CNS) tissue microarray was purchased from Zhongke Guanghua Intelligent Biotechnology Co., Ltd. (https://www.bioaitech.com/). The microarray contained 39 samples, including 8 samples with WHO grade I astrocytoma, 7 samples with WHO grade II astrocytoma, 11 samples with WHO grade III anaplastic astrocytoma, 6 samples with GBM, and 7 normal samples. Public or patient involvement in this study was not required as the samples were retrieved from Zhongke Guanghua Intelligent Biotechnology Co., Ltd. (Xi’an, China).

### Immunohistochemistry

First, the tissue microarray was dewaxed, rehydrated, and incubated in a peroxidase blocker to block the activity of endogenous peroxidase in the samples. Then, the microarray was heated with sodium citrate buffer for 15 min to repair the antigen. The nonspecific reaction of samples was blocked with goat serum and incubated with primary antibody GPX8 (Abcam, ab183664, Rabbit polyclonal, 1:500) at 4°C overnight. After the microarray was rewarmed at 37°C for 45 min, the biotin-avidin system was used for detection. Microarray was stained with hematoxylin and then differentiated using hydrochloric acid and alcohol. Finally, the sections were dehydrated, sealed with neutral resin after drying, and photographed with a microscope. Immunohistochemical staining intensity score was detected by ImageJ package IHC profiler, as high positive +4, positive +3, low positive +2, and negative +1.

### Cell culture

GBM cell lines U251, U87, and A172 were purchased from the National Collection of Authenticated Cell Culture https://www.cellbank.org.cn/), and cultured in DMEM (Hyclone) supplemented with 10% fetal bovine serum. The normal astrocytes cell line SVG p12 was purchased from American Type Culture Collection (ATCC, https://www.atcc.org/) and cultured in EMEM (ATCC) supplemented with 10% fetal bovine serum. Penicillin streptomycin was supplemented to the culture medium of all cell lines, and *mycoplasma* was detected regularly. All cell lines were grown in 5% CO_2_–95% O_2_ at 37°C, and were cultured for more than 6 months after receipt.

### Western blotting

Proteins were extracted with RIPA lysis buffer, and BCA protein analysis was used to detect the protein concentration according to the instructions. After gel electrophoresis, the blotting was transferred to a nitrocellulose membrane and blocked with 5% skim milk at RT for 1 h. Primary anti-GPX8 antibodies (1:500, 24 kDa) and β-actin (1:2000, 42 kDa) were incubated overnight at 4°C. The second antibody was labeled with horseradish peroxidase, and blotting bands were visualized using ECL. Images were acquired with iBright 1500 (Thermo Fisher Scientific) and detected by gray level using the ImageJ software.

### Immune infiltration analysis

The Tumor Immune Estimation Resource (TIMER2.0, http://timer.comp-genomics.org/) database is dedicated to the molecular characterization of tumor immune interactions, projecting immune cell infiltration subpopulations from more than 10,000 tumor samples from 32 types of cancer (Li et al., 2017). We mainly explored the expression of GPX8 in several cancers and further analyzed the correlation of GPX8 with the infiltration of immune cells including CD4^+^ T cells, CD8^+^ T cells, B cells, monocytes, macrophages, myeloid dendritic cells, NK cells, neutrophils, and tumor-associated fibroblasts in GBM.

The TISIDB (http://cis.hku.hk/TISIDB/index.php) database integrates oncoimmunology data from multiple cancers, allowing researchers to explore the functions of specific genes in tumor–immune interactions (Ru et al., 2019). The mRNA co-expression data of GPX8 with MHC genes, immunoinhibitors, and immunostimulators were obtained from the TISIDB database.

### GEPIA database analysis

The GEPIA (http://gepia.cancer-pku.cn/) database contains RNA sequencing data from large databases such as GTEx, providing a new platform for users to conduct data mining and explore gene functions (Tang et al., 2017). The expression data of GPX8 in 31 cancers compared with normal tissues was downloaded from the GEPIA database. The transcript level of GPX8 in GBM and low-grade glioma (LGG) was ascertained by the GEPIA database.

### TCGA database

The Cancer Genome Atlas (TCGA, https://portal.gdc.cancer.gov) database has been collecting molecular and clinical data for more than 33 different types of cancer since 2016, making it the largest human cancer molecular database to date (Weinstein et al., 2013). We used the R (3.7.2) package DESeq2 to compare the expression data (TCGA-GBM, HTSeq-counts) between GBM and normal tissues. The differential gene thresholds for volcano plot were |log2FC| > 3 and adjusted *p* < 0.01. Moreover, the R package corrplot was used to analyze the association between GPX8 and PMT markers.

### The Gene Expression Omnibus

The GEO database (https://www.ncbi.nlm.nih.gov/geo/) was built by NCBI to provide access to freely distributed microarray and transcriptomic data from other original studies (Barrett et al., 2013). GEO2R was used to analyze GSE67089 transcriptome data online, and by comparing GBM mesenchymal cells and GBM proneural cells, we obtained differentially expressed genes.

### The Chinese Glioma Genome Atlas

The CGGA (http://www.cgga.org.cn/) database collected and analyzed more than 2,000 glioma samples from all over China, providing users with download channels for single-cell sequencing, whole-genome sequencing, mRNA sequencing, and other datasets of glioma samples (Zhao et al., 2021). The mRNAseq 693 dataset consisting of 693 glioma tissues, including 443 LGG and 149 HGG samples, was downloaded from the CGGA database. Furthermore, the gene correlation analysis in GBM was performed using the “Correlation” module.

### The cBioPortal for Cancer Genomics

The cBioPortal for Cancer Genomics (http://cbioportal.org/) is a large cancer genomics project data integration database developed by the Memorial Sloan-Kettering Cancer Center that currently provides the public with large-scale data processing and statistical analysis at the protein to gene level (Cerami et al., 2012). cBioPortal was used to explore the co-expression genes of GPX8 in three glioma datasets and analyze the correlation between GPX8 and related genes in GBM.

### Enrichment analysis

The Metascape (https://metascape.org/) database utilizes 40 independent biological knowledge bases to systematically explain known pathways and complexes involved in genes, providing comprehensive gene annotations and resource analysis for scientific research (Zhou et al., 2019). GO analysis and functional enrichment analysis of GPX8 co-expression genes were performed by using Metascape. Gene sets with a minimum enrichment >1.5 and *p* < 0.01 were considered significant enrichment.

Gene set enrichment analysis (GSEA) was developed by the Massachusetts Institute of Technology to analyze the correlation between gene expression changes and predetermined gene sets (Subramanian et al., 2005). GSEA was used to explore the enrichment of GPX8 in the gene sets of the PMT process, and normalized enrichment score (NES) was used to quantify the degree of enrichment.

### Statistical analysis

• Log2-transformation was used to normalize the mRNA expression data. The comparison of data between two groups was performed by the t-test and Wilcoxon, and the comparison of data greater than or equal to three groups was performed by one-way analysis of variance (ANOVA). The log-rank test was used to evaluate differences in event time distribution between GPX8 expression groups. The correlation between two variables was evaluated by Pearson’s test or Spearman’s test. The Cox regression model was used to evaluate the predictive efficiency of GPX8 expression and other clinicopathological factors as independent risk factors. R software (3.7.2) and SPSS 25 software were used for statistical analysis. All of the aforementioned statistical tests were considered significant when *p* < 0.05.

## Results

### Differential expression of GPX8 in cancers

**FIGURE 1:**
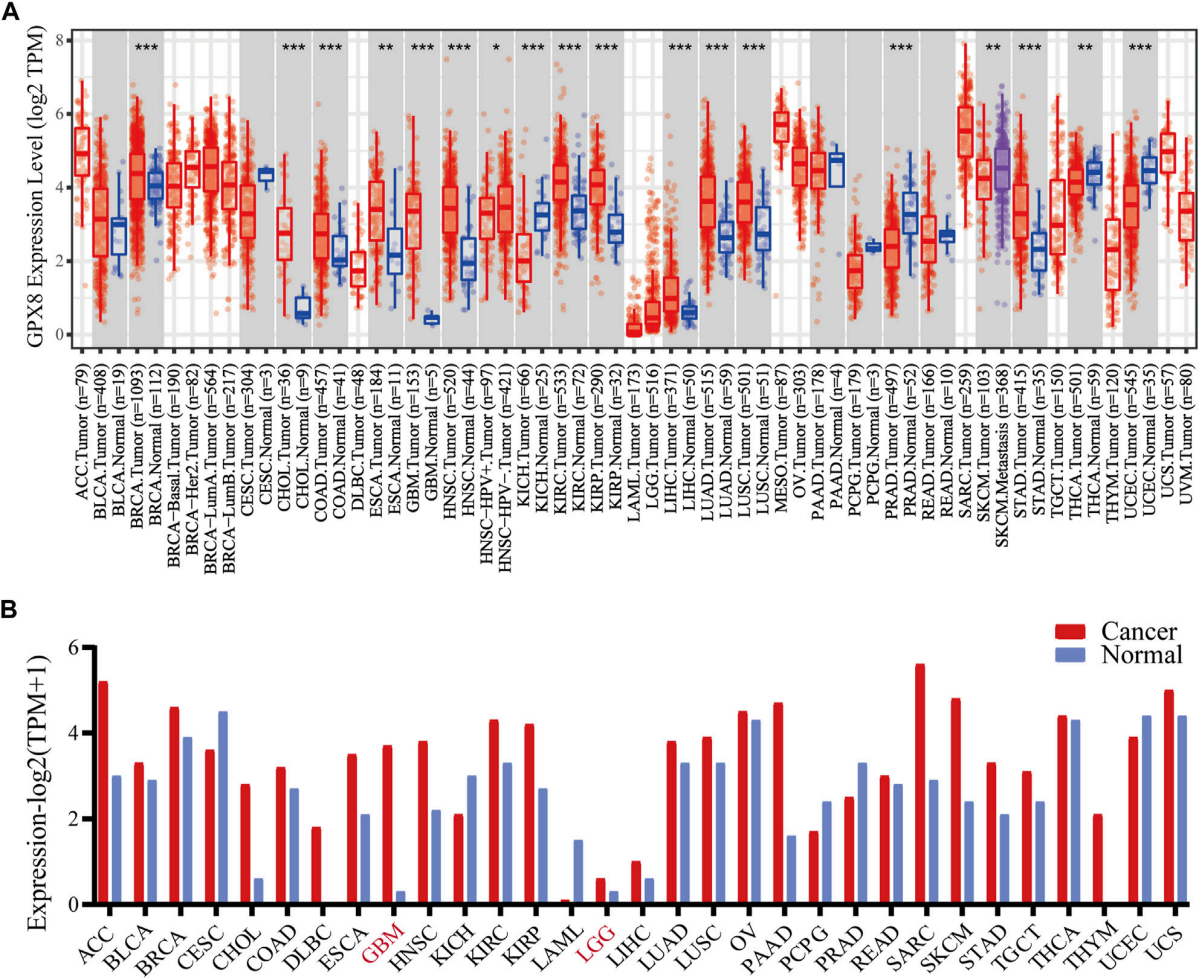
mRNA expression of GPX8 in various cancers accessing TIMER and GEPIA databases. **(A)** mRNA expression of GPX8 in cancers and homologous normal tissues from the TIMER2.0 database. **(B)** mRNA expression data of GPX8 about cancers and homologous normal tissues were obtained from the GEPIA database.*p < 0.05, **p < 0.01, and ***p < 0.001.

To evaluate the expression of GPX8 in different cancers, pan-cancer analysis was derived from TCGA by accessing the TIMER2.0 database. The analysis revealed that GPX8 was upregulated in breast, cholangio, colorectal, esophagus, kidney, liver, lung, stomach, and brain cancers compared with homologous normal tissues (Figure 1A). In addition, GPX8 was downregulated in kidney chromophobe, prostate adenocarcinoma, thyroid carcinoma, and uterine corpus endometrial carcinoma.

Moreover, we analyzed the expression of GPX8 in several cancers using the GEPIA database. According to the result, GPX8 was upregulated in the kidney, cholangio, lymph gland, esophagus, brain, pancreas, skin, stomach, and thymus cancers and downregulated in acute myeloid leukemia (Figure 1B). Combining the results of the two databases showed that GPX8 was upregulated in a variety of cancers including kidney (except chromophobe), stomach, esophagus, cholangio, and brain.

### GPX8 was highly expressed in human GBM

**FIGURE 2:**
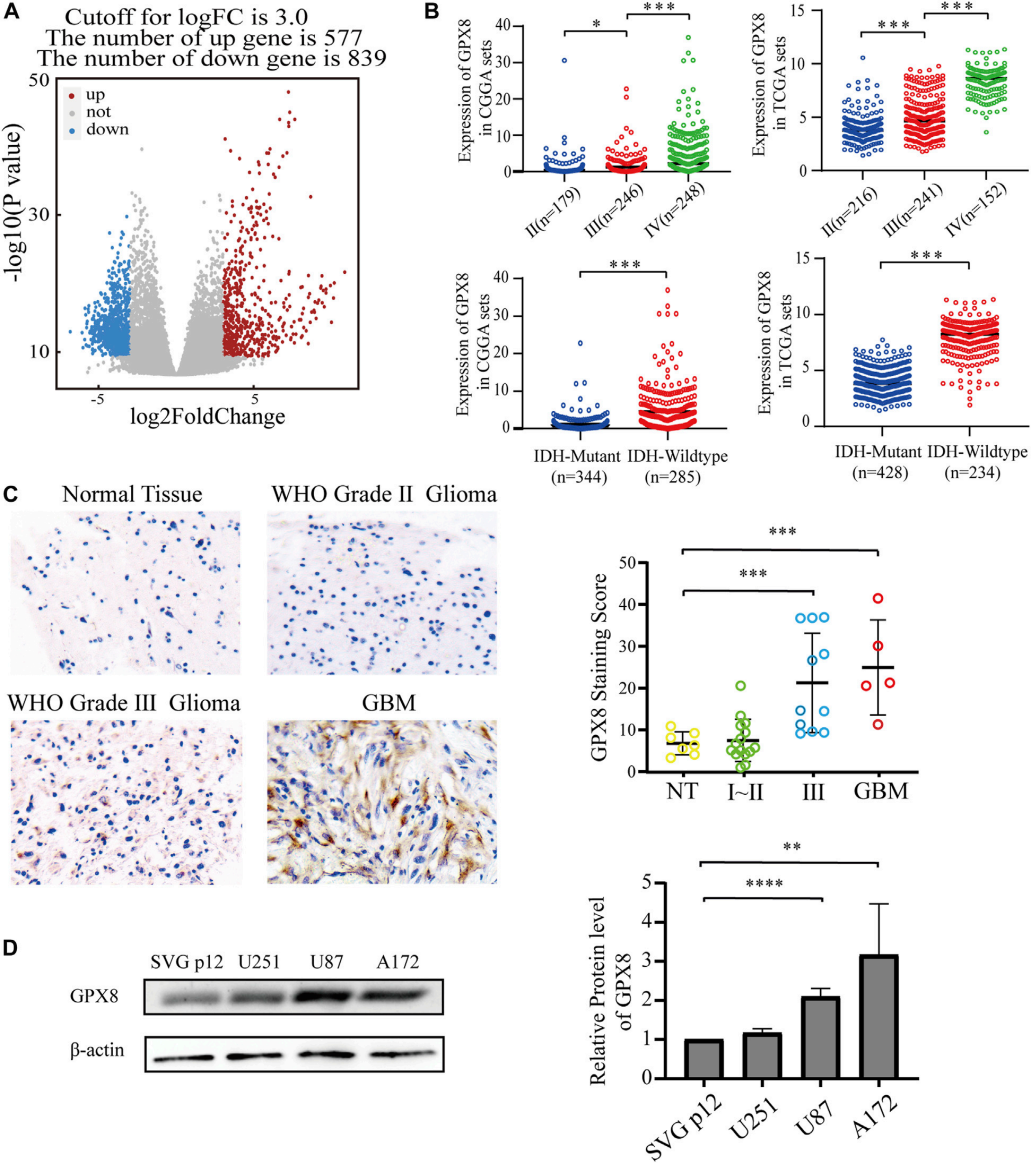
Expression of GPX8 was detected in gliomas. **(A)** Volcanic plot of DEGs of 166 GBM samples compared with five normal tissue samples, the red plot specifies upregulated genes (n = 577), and the blue plot specifies downregulated genes (n = 839). **(B)** The mRNA expression of GPX8 in gliomas, upper panel according to WHO grade, and lower panel according to IDH status. **(C,D)** Protein expression of GPX8 in gliomas. *p < 0.05, **p < 0.01, ***p < 0.001, and ****p < 0.0001.

To explore the function of GPX8 in GBM, the most aggressive malignancies of the central nervous system, we accessed the TCGA database to obtain the mRNA expression data of GBM and normal brain tissues. Next, we performed differentially expressed genes (DEGs) on the TCGA data by using the R package DESeq2, the DEGs results showed that 577 genes were upregulated and 839 genes were downregulated, with GPX8 (log2FC = 4.879635261, *p* = 4.17e-17) being one of the most significantly upregulated genes in GBM (Figure 2A). Subsequently, in order to further explore the function of GPX8, we obtained the mRNA expression data of GPX8 in glioma by accessing CGGA and TCGA databases. Noticeably, the mRNA expression of GPX8 was correlated with glioma WHO grade (Figure 2B upper panel). Moreover, in both of TCGA and CGGA datasets, GPX8 had higher expression in IDH-wildtype gliomas (Figure 2B lower panel).

We then explored the protein levels of GPX8 in glioma specimens. GPX8 showed higher expression in tumors than in normal brain tissues, especially in HGG (Figure 2C). We also performed a western blot for GPX8 expression in GBM cell lines and normal astrocyte cell lines. GPX8 was overexpressed in GBM cell lines compared with normal astrocyte cell lines (Figure 2D). Collectively, these results suggest that the expression of GPX8 may be associated with the malignant progression of glioma.

### The upregulation of GPX8 correlated with poor prognosis in gliomas

**FIGURE 3:**
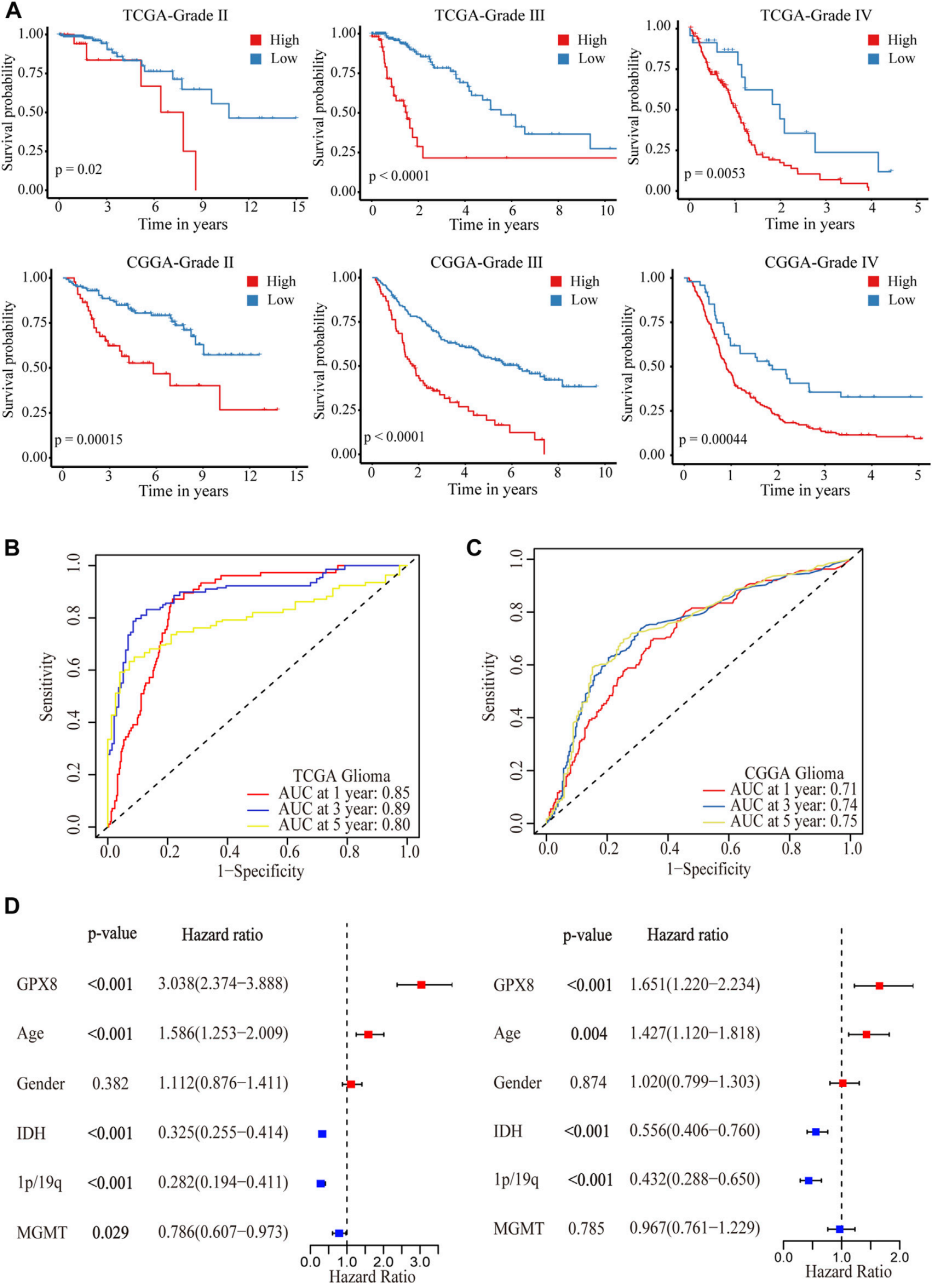
Correlation analysis between GPX8 and prognosis of glioma patients. **(A)** Kaplan–Meier overall survival plot showing survival rates for all WHO-grade glioma patients with GPX8 low expression (blue), and high expression (red) in TCGA and CGGA datasets. **(B,C)** Time-dependent ROC analysis showing the prognostic efficacy of GPX8 for glioma patients in TCGA and CGGA datasets. **(D)** Forest plot for univariate and multivariate Cox analysis of CGGA data.

After showing the expression of GPX8 in gliomas, we are interested in GPX8 affecting the clinical outcome in glioma patients. We queried TCGA and CGGA datasets, and found that the expression of GPX8 was associated with poor prognosis in WHO grade Ⅱ, Ⅲ, and Ⅳ glioma patients, as shown in Figure 3A. Moreover, the upregulation of GPX8 in both primary and recurrent gliomas affected the prognosis of patients (Supplementary Figure S1).

To further verify the prognostic efficacy of GPX8 for glioma patients, ROC analysis was performed on TCGA and CGGA data. The 1-year, 3-year, and 5-year AUC of TCGA time-dependent ROC were 0.85, 0.89, and 0.80 (Figure 3B), and those of CGGA were 0.71, 0.74, and 0.75 (Figure 3C), respectively. Furthermore, univariate and multivariate Cox analyses were performed on the CGGA OS data of HGG patients. As shown in Figure 3D, the univariate analysis showed that GPX8 (HR = 3.038, *p* < 0.001), age (HR = 1.586, *p* < 0.001), IDH-mutation (HR = 0.325, *p* < 0.001), 1p19q co-deletion (HR = 0.282, *p* < 0.001), and MGMT promoter methylation (HR = 0.786, *p* = 0.029) are the prognostic factors for OS of the HGG. Similarly, the multivariate analysis showed that GPX8 (HR = 1.651, *p* = 0.001), age (HR = 1.427, *p* = 0.004), IDH-mutation (HR = 0.556, *p* < 0.001), and 1p19q co-deletion (HR = 0.432, *p* < 0.001) are the prognostic factors for OS of the HGG patients. Collectively, these results indicated that GPX8 is an independent prognostic factor for the OS of glioma patients.

### GPX8 correlated with the PMT process of GBM

**FIGURE 4:**
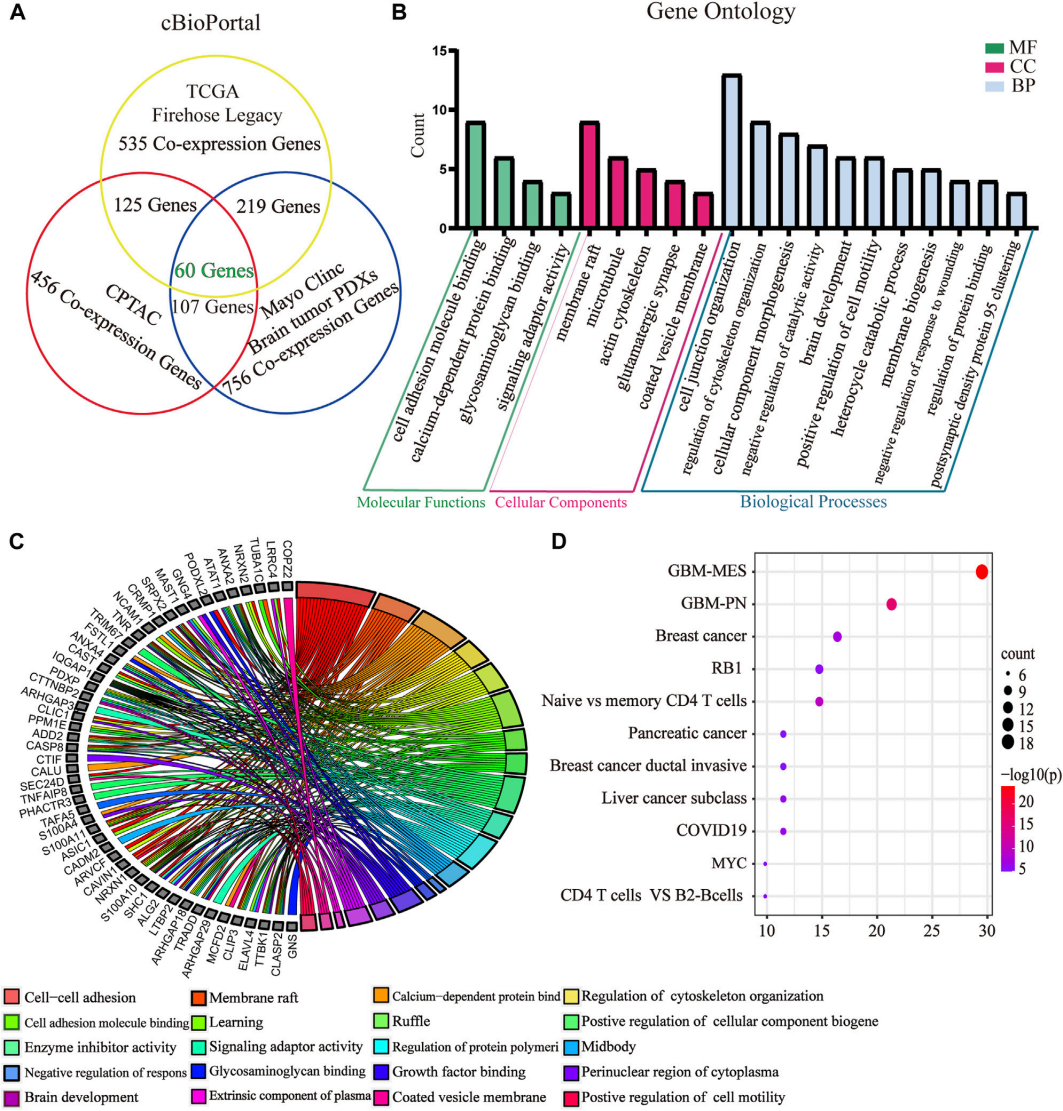
GO and function enrichment analysis of GPX8-related genes. **(A)** GPX8 expression–related genes were screened by the cBioPortal database. Screening condition is cor > |0.500| and *p* < 0.05. **(B)** GO analysis of GPX8 and related genes using the Metascape database (*p* < 0.05). **(C)** Chord plot of the GO analysis. Colors specify different biological processes. **(D)** Function enrichment analysis of GPX8 and related genes using the Metascape database.

To further evaluate the role of GPX8 in the malignancy of GBM, we obtained GPX8 expression–related genes from three datasets by accessing the cBioPortal database. Combining the results of the three datasets, we finally identified 60 genes related to GPX8 expression in GBM (Figure 4A) and validated those genes using the CGGA dataset (Table 1). Next, GO analysis was performed on the data that showed significant enrichment in common biological processes related to EMT (Figures 4B,C). Furthermore, function enrichment analysis revealed GPX8 to be significantly associated with the mesenchymal GBM and proneural GBM (Figure 4D). These results suggest that GPX8 may impact the progression of GBM by promoting PMT.

**TABLE 1:** Correlation analysis of GPX8 and related genes in primary GBM samples (*n* = 140) from the CGGA database.

Gene	Cor	*p*	Gene	Cor	*p*
COPZ2	0.568	2.07e-10	LRRC4	−0.626	7.56e-13
TUB1AC	0.547	5.94e-28	XRXN2	−0.457	8.41e-07
ANXA2	0.804	3.21e-25	ATAT1	−0.311	1.18e-03
DPF1	−0.455	9.68e-07	MAST1	−0.598	1.25e-11
PODXL2	−0.524	7.99e-09	DPYD	0.730	6.52e-19
GNG4	−0.417	8.87e-06	OLIG1	−0.591	2.46e-11
SRPX2	0.772	3.18e-22	CRMP1	−0.368	1.06e-04
PPIC	0.755	9.35e-21	NCAM1	−0.493	7.83e-08
TNR	−0.549	1.09e-09	TRIM67	−0.505	3.40e-08
FSTL1	0.778	1.00e-22	ANXA4	0.486	1.26e-07
CAST	0.685	5.25e-16	IQGAP1	0.681	9.49e-16
PDXP	−0.660	1.46e-14	CTTNBP2	−0.512	1.98e-18
ARHGAP33	−0.341	3.42e-04	CLIC1	0.724	1.68e-18
PPM1E	−0.193	4.70e-02	ADD2	−0.383	5.08e-05
GDAP1L1	−0.551	8.99e-10	CASP8	0.596	1.59e-11
CTIF	−0.498	5.58e-08	ELAVL3	−0.314	1.06e-03
CALU	0.676	1.97e-15	SEC24D	0.633	3.51e-13
TNFAIP8	0.727	1.19e-18	PHACTR3	−0.736	2.35e-19
TAFA5	−0.609	7.78e-10	FAM114A1	0.683	8.10e-13
S1004	0.638	1.91e-13	S100A11	0.604	7.09e-12
ASIC1	−0.491	9.30e-08	CADM2	−0.494	7.38e-08
ARVCF	0.256	7.98e-03	CAVIN1	0.816	3.01e-21
NRXN1	−0.512	2.01e-08	S100A10	0.606	5.74e-12
FNDC3B	0.701	6.11e-17	SHC1	0.698	8.57e-17
ALG2	0.567	2.34e-10	LTBP2	0.675	2.22e-15
ARHGAP18	0.711	1.45e-17	SEZ6L	−0.567	2.33e-10
MCFD2	0.350	2.33e-04	CLIP3	−0.661	1.00e-11
TRADD	0.430	4.12e-06	ARHGAP29	0.539	2.54e-09
ELAVL4	−0.511	2.11e-08	TTBK1	−0.473	3.08e-07
CLASP2	−0.439	2.56e-06	GNS	0.440	2.34e-06

**FIGURE 5:**
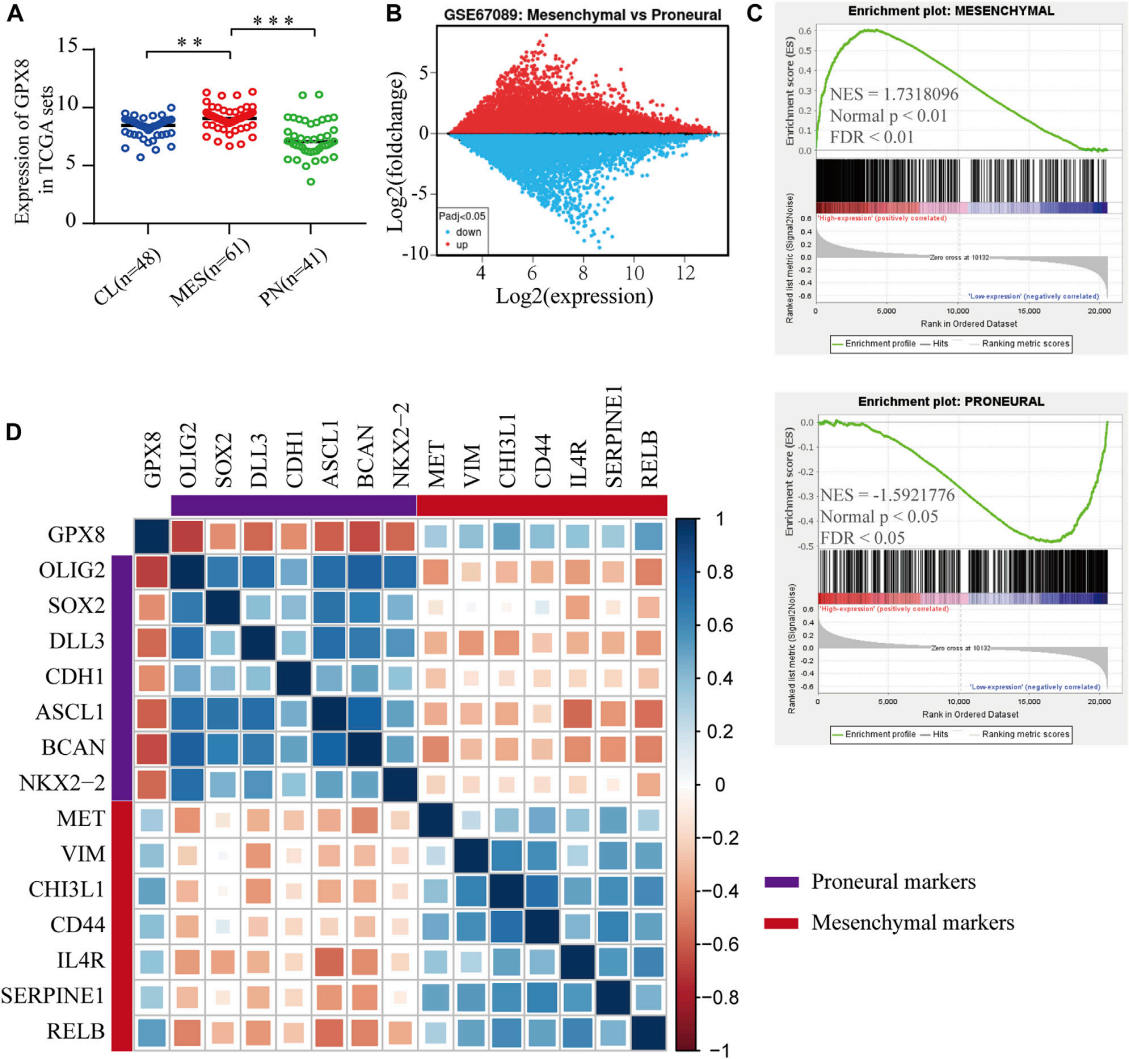
Upregulation of GPX8 was associated with the development of PMT. **(A)** The mRNA expression of GPX8 according to GBM subtypes in the TCGA-GBM set (n = 150). **(B)** Expression of GPX8 in GSC samples of the GSE67089 dataset. Red plot specifies upregulated genes, blue plot specifies downregulated genes. **(C)** GSEA plots showed that the mRNA expression of GPX8 was associated with the mesenchymal and proneural signatures. **(D)** Correlation analysis of mRNA expression of GPX8 and PMT-related gene markers in TCGA-GBM sets (n = 152). The size of the square shows the *p*-value, and the color intensity of each square represents the correlation coefficient. *p < 0.05, **p < 0.01, and ***p < 0.001.

To elucidate the function of GPX8 in PMT process, we explored the mRNA expression of GPX8 in GBM subtypes, which showed that the expression of GPX8 in mesenchymal subtype was higher than that in the proneural subtype and classical subtype (Figure 5A). Next, GEO2R analysis was performed on the GSE67089 dataset to compare DEGs between mesenchymal glioblastoma stem cells (GSCs) and proneural GSCs. Similarly, we found that GPX8 was upregulated in mesenchymal GSCs (log2FC = 2.05896662, log2Exp = 6.845, and *p* = 5.63e-05), as shown in Figure 5B. Furthermore, GSEA was further used to analyze mesenchymal and proneural signatures based on the TCGA-GBM dataset (n = 152). As Figure 5C upper panel shows, the gene signature of mesenchymal was enriched in GPX8 high expression GBM compared with GPX8 low expression GBM (NES = 1.7318096, *p* < 0.01, and FDR <0.01). In contrast, the upregulation of GPX8 in GBM was negatively correlated with proneural signature enrichment (NES = -1.5921776, *p* < 0.05, and FDR <0.05), as shown in Figure 5C lower panel. We then examined the correlation of GPX8 with mesenchymal and proneural markers using TCGA-GBM datasets (n = 152). GPX8 was positively correlated with the expression of mesenchymal markers MET (cor = 0.33), VIM (cor = 0.38), CHI3L1 (cor = 0.49), CD44 (cor = 0.40), IL4R (cor = 0.36), SERPINE1 (cor = 0.34), RELB (cor = 0.52), and negatively correlated with the expression of proneural markers OLIG2 (cor = −0.68), SOX2 (cor = −0.45), CDH1 (cor = −0.45), ASCL1 (cor = −0.59), BACN (cor = −0.64), and NKX2-2 (cor = −0.56), all *p* < 0.001 (Figure 5D). Based on these results, it is clear that GPX8 plays a pivotal role in the development of PMT.

### Correlation of GPX8 with immune infiltration in GBM

**FIGURE 6:**
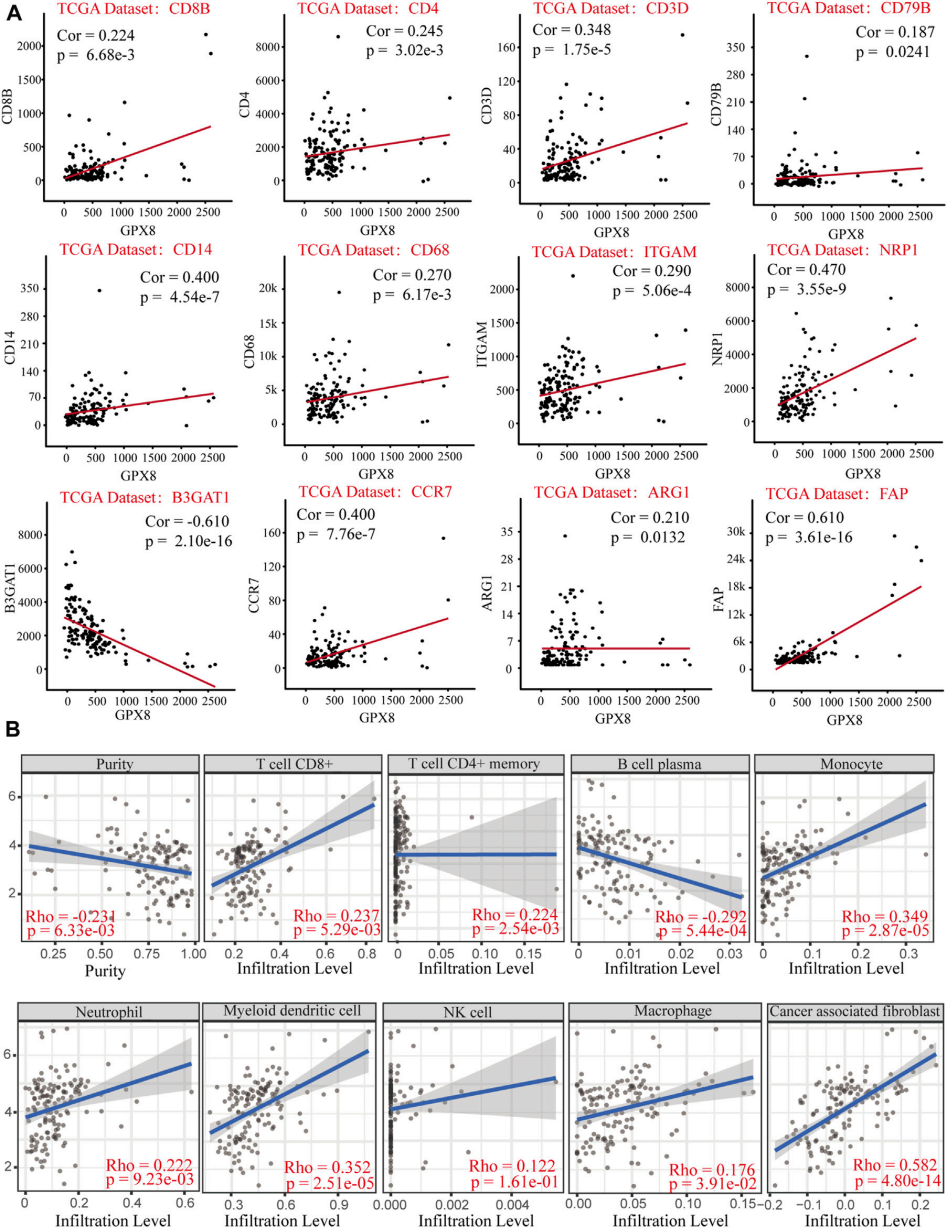
Correlation analysis of GPX8 mRNA expression with immune infiltration based on cBioPortal and TIMER2.0 databases. **(A)** Correlation between GPX8 and immune cell markers based on the cBioPortal database. **(B)** Correlation between GPX8 mRNA expression and immune cells infiltration accessing the TIMER2.0 database.

Relationship of GPX8 mRNA expression with immune markers in GBM was accessed using correlation analysis from the cBioPortal database. GPX8 mRNA expression was weakly (cor <0.2) correlated with B-cell marker CD79B and moderately (0.2 < cor <0.4) correlated with CD8^+^ T-cell marker CD8B, CD4^+^ T-cell marker CD4, T-cell marker CD3D, macrophage marker CD68, ARG1, and neutrophil marker ITGAM (Figure 6A). Meanwhile, GPX8 mRNA expression was significantly correlated with monocyte marker CD14, dendritic-cell marker NRP1, natural killer cell marker B3GAT1, neutrophil marker CCR7, and tumor-associated fibroblast marker FAP (Figure 6A). In addition, to further clarify tumor immune function of GPX8, we then analyzed the correlation between GPX8 mRNA expression and levels of immune cell infiltration in GBM using the TIMER2.0 database. The result showed that GPX8 expression was positively correlated with infiltration of CD8^+^ T cells (rho = 0.237, *p* = 5.29e-03), CD4^+^ T cells (rho = 0.224, *p* = 2.54e-03), monocytes (rho = 0.349, *p* = 2.87e-05), neutrophils (rho = 0.222, *p* = 9.23e-03), myeloid dendritic cells (rho = 0.352, *p* = 2.51e-05), macrophages (rho = 0.173, *p* = 3.91e-02), and tumor-associated fibroblasts (rho = 0.582, *p* = 4.80e-14), and negatively correlated with plasma cell (rho = −0.292, *p* = 5.44e-04) infiltration in the GBM microenvironment (Figure 6B).

**FIGURE 7:**
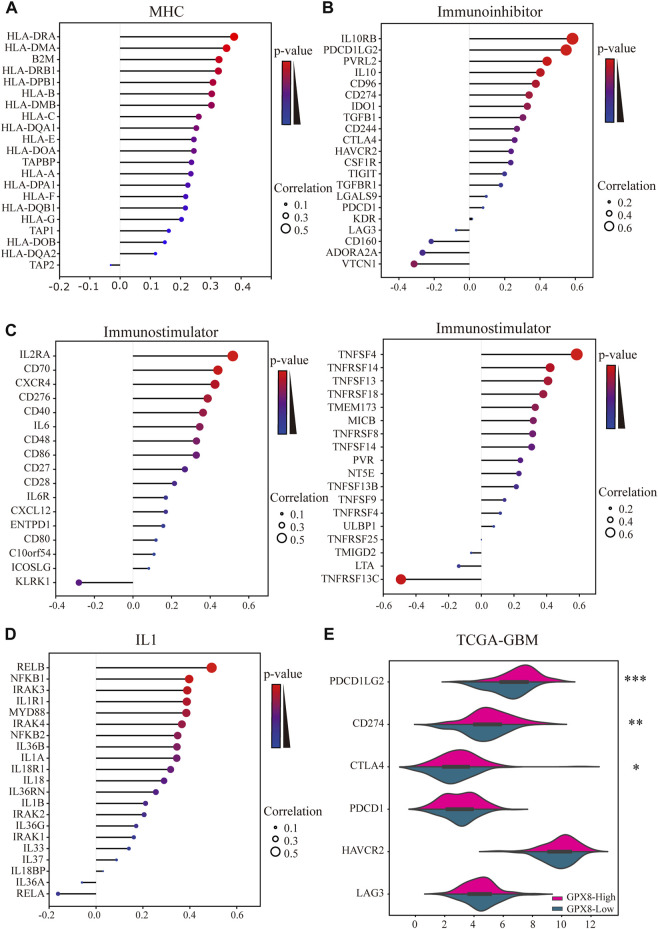
Results of analysis between the expression of GPX8 and immune markers. Co-expression of GPX8 with **(A)** MHC molecules, **(B)** immunoinhibitors, **(C)** immunostimulators, and **(D)** IL1 pathway–related molecules based on TISIDB and cBioPortal databases. **(E)** Different expression of immune checkpoints in GBM with high or low expression of GPX8. *p < 0.05, **p < 0.01, and ***p < 0.001.

Moreover, we comprehensively elucidated the correlation of GPX8 and the immune molecules in GBM by using TISIDB and cBioportal databases. MHC molecules, immunoinhibitors, immunostimulators, and IL1 pathway–related molecules were assessed. Our results showed that the mRNA expression of GPX8 was positively correlated with MHC molecules except for HLA-DOB, HLA-DQA2, and TAP2 (Figure 7A). Furthermore, GPX8 was positively correlated with almost all immunoinhibitors, but negatively correlated with VTCN1, ADORA2A, and CD160 (Figure 7B). As shown in Figure 7C, we also found that GPX8 had a positive relationship with most of the immunostimulators. Notably, except for no relation with IL33, IL18BP, IL36A, IL37, IRAK1, and RELA, the mRNA expression of GPX8 was significantly positively correlated with almost all IL1 families and MYD88-IRAK-NF-κB axis molecules (Figure 7D). Based on the TCGA-GBM dataset, we further explored the expression of immune checkpoints, including PDCD1, PDCD1LG2, CD274, CTAL4, HAVCR2, and LAG3, and found that PDCD1LG2 (*p* < 0.0001), CD274 (*p* = 0.0011), and CTLA4 (*p* = 0.011) were upregulated in GBM patients with a higher expression of GPX8 (Figure 7E). Collectively, our findings revealed that GPX8 plays a pivotal role in the immune infiltration of the GBM tumor microenvironment.

## Discussion

GBM, a heterogeneous disease, is the most common primary solid brain cancer (Brennan et al., 2013). GBM cells invaded adjacent brain tissues, resulting in tumor recurrence and therapy resistance, but metastasis rarely occurred (Romo et al., 2019). In addition to its unique tumor cell subgroups, the GBM microenvironment contains a variety of nonneoplastic cell subgroups, which interact with other cells in complicated ways, forming an ecological environment that affects the growth of GBM (Chen and Hambardzumyan, 2018). Investigating the molecular mechanism of the interaction between different cell subgroups in GBM has significant implications for therapy.

Among the molecular types of GBM, the proneural type commonly exhibits the amplification of PDGFRA loci, and the mesenchymal type is characterized by NF1 mutation (Neftel et al., 2019). GBM therapy resistance and recurrence were correlated with the transition of cells to mesenchymal phenotype (Li et al., 2017). This transition seems to be achieved by molecular interactions between different cell phenotypes. For example, malic enzyme 2 (ME2) inhibited the proneural marker OLIG2 and upregulated mesenchymal markers to drive PMT (Yang et al., 2021). Interestingly, a recent study showed that proneural marker ASCL1 maintained the proneural phenotype of tumor cells by inhibiting the expression of mesenchymal marker NDRG1 (Narayanan et al., 2019). The transition was correlated with the plasticity of GBM cells. GPX8 was described as similar to the known mesenchymal markers (Shaul et al., 2016). Our data showed that GPX8 was enriched in EMT-related progressions such as regulation of cytoskeleton organization and cell adhesion molecule binding (Yilmaz and Christofori, 2009; Wang et al., 2018). Furthermore, we found that GPX8 expression was positively correlated with mesenchymal markers and negatively correlated with proneural markers (Figure 5D). These data suggest that GPX8 overexpression promoted GBM malignancy by driving PMT. The direct evidence showing GPX8 was involved in PMT is still absent, but our study provides valuable clues. The phenotypes transition vulnerability has significant effects on the progression of GBM.

Immunotherapy, the crucial part of clinical tumor therapy, has the potential to conquer GBM. The blood–brain barrier (BBB) and cellular heterogeneity were the main obstacles to immunotherapy for GBM (Jackson et al., 2019). BBB intercepts hydrophilic molecules and reduces the immune response of CNS (Bauer et al., 2014). The GBM microenvironment contains some nonneoplastic cells from the immune system. These nonneoplastic cells secreted cytokines that inhibited immune responses and promoted the progression of GBM (Chen and Hambardzumyan, 2018). It has been reported that GPX8, expressed in macrophages, may be involved in immune defense (Hsu et al., 2020). Correlation analysis was performed to explore the connections between GPX8 and immune markers. Our data demonstrated that GPX8 was correlated with mRNA expression of immune markers of CD8^+^ T cells (CD8B), CD4^+^ T cells (CD4), T cells (CD3D), macrophages (CD68 and ARG1), neutrophils (ITGAM and CCR7), dendritic cells (NRP1), NK cells (B3GAT1), and tumor-associated fibroblasts (FAP). Meanwhile, the expression of GPX8 was associated with the infiltration of CD8^+^ T cells, tumor-associated fibroblasts, plasma cells, macrophages, myeloid dendritic cells, neutrophils, and CD4^+^ T cells in the GBM microenvironment, but not with NK cells. Moreover, we also found that the expression of MHC molecules, immunoinhibitors, and immunostimulators were correlated with GPX8 in GBM (Figures 7A–C). These results suggest that GPX8 may inhibit tumor immune response by recruiting immune cell infiltration.

IL1 family members are key molecules mediating adaptive and innate immunity, and they signal via the MyD88-IRAK-NFκB pathway (O'Neill, 2002). IL1 can recruit immune cell infiltrates to reshape the tumor microenvironment (Mantovani et al., 2018), and targeting IL1 may benefit some clinical cancer patients (Hong et al., 2014). Here, we performed a correlation analysis to investigate the connections between GPX8 and IL1-related molecules. In GBM samples, GPX8 was correlated with the expression of IL1A, IL1B, IL1R1, IL18, IL18R1, IL36B, IL36G, IL36RN, MYD88, IRAK2, IRAK3, IRAK4, NFKB1, NFKB2, and RELB, as shown in Figure 7D.

To our knowledge, this is the first study to explore the biological function of GPX8 in GBM. GPX8 has received extensive attention recently, especially in cancer research (Zhang et al., 2020; Chen et al., 2020; Khatib et al., 2020), but its function remains unclear. Our study shows that the mRNA and protein expression of GPX8 were upregulated in glioma. Notably, the expression of GPX8 in HGG was significantly higher than that in LGG suggesting that malignant progression of glioma was correlated with GPX8 overexpression. In addition, GPX8 overexpression was associated with poor prognosis in patients with primary or recurrent gliomas, as shown in Supplementary Figure S1. Finally, we demonstrated that GPX8 is a potential target for immunotherapy and participates in GBM phenotype transition.

## Data Availability

The original contributions presented in the study are included in the article/Supplementary Material; further inquiries can be directed to the corresponding authors.
