# Supplementary material for: The overexpression of GPX8 is correlated with poor prognosis in GBM patients
Source: Front Genet. 2022 Aug 17;13:898204. doi: 10.3389/fgene.2022.898204 (PMC9432423; doi:10.3389/fgene.2022.898204)
Supplement: Supplementary file 1 [file Table1.DOCX]

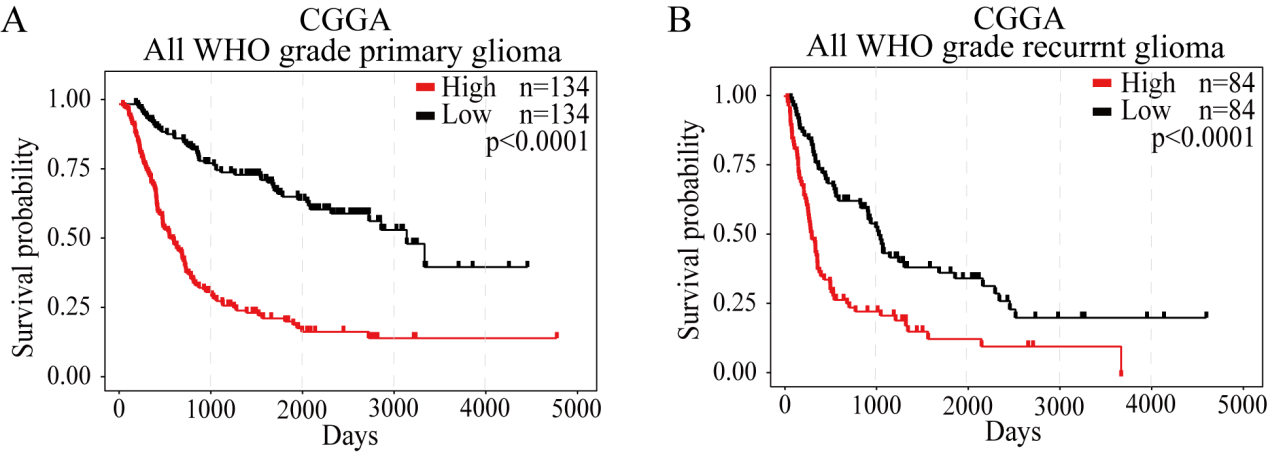


**FIGURE S1** **|** Survival analysis of all WHO grade glioma based on CGGA database. **(A)** Kaplan–Meier overall survival plot showing survival rates for primary glioma patients . **(B)** Kaplan–Meier overall survival plot showing survival rates for recurrnt glioma patients.
